# Investigating the Association Between the Caecal Microbiomes of Broilers and *Campylobacter* Burden

**DOI:** 10.3389/fmicb.2018.00927

**Published:** 2018-05-22

**Authors:** Ioannis Sakaridis, Richard J. Ellis, Shaun A. Cawthraw, Arnoud H. M. van Vliet, Dov J. Stekel, Johanna Penell, Mark Chambers, Roberto M. La Ragione, Alasdair J. Cook

**Affiliations:** ^1^School of Veterinary Medicine, University of Surrey, Guildford, United Kingdom; ^2^Animal & Plant Health Agency, Weybridge, United Kingdom; ^3^School of Biosciences, University of Nottingham, Nottingham, United Kingdom; ^4^Department of Clinical Sciences, Swedish University of Agricultural Sciences, Uppsala, Sweden

**Keywords:** caecal microbiomes, broilers, *Campylobacter* infections, sequencing, microbial community

## Abstract

One of the major transmission routes for the foodborne bacterial pathogen *Campylobacter* is undercooked poultry meat, contaminated from intestinal contents during processing. In broilers, *Campylobacter* can grow to very high densities in the caeca, and is often considered to be a commensal or an opportunistic pathogen in poultry. Reduction of caecal loads of *Campylobacter* may assist in lowering incidence rates of *Campylobacter* food poisoning. To achieve this, there needs to be a better understanding of the dynamics of *Campylobacter* colonization in its natural niche, and the effect of the local microbiome on colonization. Previous studies have shown that the microbiome differed between *Campylobacter* colonized and non-colonized chicken intestinal samples. To characterize the microbiome of *Campylobacter*-colonized broilers, caecal samples of 100 randomly selected birds from four farms were analyzed using amplified 16S rRNA gene sequences. Bacterial taxonomic analysis indicated that inter-farm variation was greater than intra-farm variation. The two most common bacterial groups were Bacteroidetes and Firmicutes which were present in all samples and constituted 29.7–63.5 and 30.2–59.8% of the bacteria present, respectively. *Campylobacter* was cultured from all samples, ranging from 2 to 9 log_10_ CFU g^-1^. There was no clear link between *Campylobacter* counts and Firmicutes, Bacteroidetes, or Tenericutes levels in the 16S rRNA operational taxonomic unit (OTU)-based analysis of the caecal microbiome, but samples with high *Campylobacter* counts (>9 log CFU g^-1^) contained increased levels of *Enterobacteriaceae*. A decrease in *Lactobacillus* abundance in chicken caeca was also associated with high *Campylobacter* loads. The reported associations with *Lactobacillus* and Enterobacteriaceae match changes in the intestinal microbiome of chickens and mice previously reported for *Campylobacter* infection, and raises the question about temporality and causation; as to whether increases in *Campylobacter* loads create conditions adverse to Lactobacilli and/or beneficial to Enterobacteriaceae, or that changes in Lactobacilli and Enterobacteriaceae levels created conditions beneficial for *Campylobacter* colonization. If these changes can be controlled, this may open opportunities for modulation of chicken microbiota to reduce *Campylobacter* levels for improved food safety.

## Introduction

The pathogenic *Campylobacter* species *Campylobacter* *jejuni* and *Campylobacter* *coli* are common causative agents of bacterial gastroenteritis in humans (Nichols et al., 2012). One of the primary transmission routes is via ingestion of undercooked meat, especially poultry (Tam et al., 2009, 2012). During processing, caecal contents can contaminate the surface of the meat. If the bacteria survive further processing and food preparation, they can cause diarrheal disease and are also implicated in post-infectious sequelae such as Guillain–Barré syndrome (Hughes, 2005).

The presence of *Campylobacter* spp. in chicken carcasses is an important risk factor for food borne campylobacteriosis. Foodborne *Campylobacter* is the major cause of bacterial food poisoning with an estimated 280,000 cases each year in the United Kingdom (FSA, 2016). According to EFSA, up to 80% of cases can be attributed to contaminated poultry meat and a 10-fold decrease in the exposure levels from this source is likely to reduce the number of human *Campylobacter* cases by 50 to 90% across all Member States (EFSA, 2011). An EU baseline survey including data from 26 European Union Member States and two countries not belonging to the European Union showed that 75% of broiler batches carried *Campylobacter* in their caecal contents and 86% of skin samples were contaminated (EFSA, 2010). Moreover, the United Kingdom Food Standards Agency (FSA) reported that 61.3% of fresh, United Kingdom-produced, retail samples were contaminated (Jorgensen et al., 2017). Aiming to reduce the prevalence of *Campylobacter* in chicken, FSA has set up a joint target with industry to reduce contamination levels to less than 1000 colony forming units per gram of chicken skin (CFU g^-1^) because it is believed that chickens with this level, or higher, of *Campylobacter* contamination are the most likely to infect consumers (EFSA, 2011).

Reducing the prevalence of *Campylobacter* contamination in carcasses and in retail products could be achieved by decreasing the on-farm prevalence either by reducing the number of infected flocks and/or reducing levels within individual birds. Although several biosecurity measures have been suggested to reduce the occurrence of *Campylobacter* at poultry farms, including appropriate hygiene barriers, the use of house-specific boots and clothes, the use of overshoes and the effective use of boot dips, these have not been consistently successful (Fonseca et al., 2016). Once *Campylobacter* is present there is rapid transmission within the flock, which may be exacerbated by factors including stocking density and litter conditions (Conlan et al., 2007; Torok et al., 2009; Collett, 2012). The ability of a given strain to colonize the broiler gut will be dependent on the strain characteristics, the immune status and health of the bird and the composition of the existing microbiological population – the gut microbiome (Qu et al., 2008). The microbiome of individual birds, the flock and the environment can be considered as a single ecological system that will influence the occurrence, incidence, and burden of infection (Robinson et al., 2010). Through gaining detailed knowledge of the microbiome and of *Campylobacter* colonization we hope to inform interventions aimed at reducing the prevalence of *Campylobacter* in broilers. For example, through the informed use of probiotic bacteria. However, knowledge underpinning a rationale use of probiotics is currently limited.

Modern sequencing approaches are currently used to characterize the intestinal microbiome composition of broilers. Considering that all birds within a flock have the same age, feed, and similar genetics it may be expected that the profiles of individual birds within a flock would be similar. However, a recent study has shown that there is high bird to bird variation, within the same flock (Thibodeau et al., 2015). Knowledge of the magnitude of microbiome genetic variation within and between flocks is still lacking.

The overall aim of this study was to characterize the microbial communities of the caeca of broiler chickens in relation to the prevalence and burden of *Campylobacter* infection. It is generally accepted that caeca are the predominant sites for colonization of *Campylobacter* in broilers and the *Campylobacter* load in the caecum is an important risk factor for carcass contamination of commercial birds (Hermans et al., 2011). To accomplish the goals of this study we characterized the microbiome of 100 individual birds from four different flocks; compared individual samples in order to determine the intra-flock and inter-flock variation in the relative abundance of bacterial genera within the caeca; explored the association between presence and abundance of *Campylobacter* and other genera within the samples.

## Materials and Methods

### Sample Collection and Preparation of DNA Samples

Four batches (each *n* = 25) of carcasses of conventionally reared broiler chickens (**Table 1**) were taken from a poultry processing plant and transported to the Animal and Plant Health Agency (APHA) on the day of slaughter. Caeca were removed whole from the carcasses and a sample of the contents processed immediately for quantitative bacteriology (see below). An entire caecum and contents from each carcass were stored at -20°C until required. Caeca were left to thaw for 1 h at room temperature and their contents transferred to sterile universals and homogenized by stirring with sterile plastic sticks. Caecal contents (25 mg) were transferred to sample tubes for DNA extraction. Community DNA was isolated from the 100 individual caecal samples using Mo Bio Power Soil DNA kits (now DNeasy PowerSoil, Qiagen), following the manufacturer’s protocol.

**Table 1:** Age and average weight of all birds grouped by farms.

Farm	Date visit	Age (d)	Mean weight (g)	Weight range (g)
Farm 1	16/06/2014	34	1,595.12	1,117–2,101
Farm 2	09/06/2014	35	1,648.72	1,248–1,995
Farm 3	02/06/2014	34	2,057.96	1,618–2,355
Farm 4	20/05/2014	39	NR	NR


*NR, not recorded.*

### *Campylobacter* Culture

The levels of *Campylobacter* in all 100 birds were determined by quantitative bacteriology, as described previously (Wassenaar et al., 1993). Briefly, fresh caecal contents were suspended in sterile PBS (1/10, w/v) and serial dilutions plated onto 5% sheep’s blood agar plates containing Skirrow’s Campylobacter Selective Supplement (Oxoid) and cefoperazone (20 μg/ml; Sigma). Plates were incubated in a microaerobic incubator (Heraeus) (Gas composition: 8% O_2_, 7.5% CO_2_, 84.5% N_2_) at 42°C for 48 h.

### PCR Amplification and rRNA Gene Sequencing

Aliquots of extracted DNA were amplified with universal primers for the V4 and V5 regions of the 16S rRNA gene. The primers U515F (5′-GTGYCAGCMGCCGCGGTA) and U927R (5′-CCCGYCAATTCMTTTRAGT) (Ellis et al., 2013) were designed to permit amplification of both bacterial and archaeal ribosomal RNA gene regions. Forward and reverse fusion primers consisted of the Illumina overhang forward (5′-TCGTCGGCAGCGTCAGATGTGTATAAGAGACAG) and reverse adapter (5′-GTCTCGTGGGCTCGGAGATGTGTAATAAGAGACAG) respectively. Amplification was performed with FastStart HiFi Polymerase (Roche Diagnostics, Ltd., United Kingdom) using the following cycling conditions: 95°C for 3 min; 25 cycles of 95°C for 30 s, 55°C for 35 s, 72°C for 1 min; followed by 72°C for 8 min. Amplicons were purified using 0.8 volumes of Ampure XP magnetic beads (Beckman Coulter). Each sample was then tagged with a unique pair of indices and the sequencing primer, using Nextera XT v2 Index kits, and 2x KAPA HiFi HotStart ReadyMix using the following cycling conditions: 95°C for 3 min; 12 cycles of 95°C for 30 s, 55°C for 30 s, 72°C for 30 s; followed by 72°C for 5 min. Index-tagged amplicons were purified using 0.8 volumes of Ampure XP magnetic beads (Beckman Coulter). The concentration of each sample was measured using the fluorescence-based Quantifluor assay (Promega). Concentrations were normalized before pooling all samples, each of which would be subsequently identified by its unique index combination. Sequencing was performed on an Illumina MiSeq with 2 × 300 base reads according to the manufacturer’s instructions (Illumina, Cambridge, United Kingdom).

### Data Analysis

Sequence reads obtained were processed according to the microbiome-helper pipeline^1^. Essentially paired end reads were merged based on overlapping ends using PEAR^2^, before filtering the data for base-calling quality and amplicon length. The processed sequences were then classified using the pick open reference OTUs process implemented in QIIME v1.9.1 (Caporaso et al., 2010) against the Greengenes 16S rRNA gene database^3^ using a 97% similarity cut-off. The resulting distribution of OTUs across the multiple samples was further analyzed using QIIME v1.9.1 to summarize the distributions and explore alpha (number of different types of sequences in a sample) and beta (how different types are distributed among samples) diversity. Individual samples were assigned to specific categories to explore intra-farm differences and the relationship between *Campylobacter* load and the composition of the microbiome. Relative abundance plots of microbial taxa were generated to visualize the differences between categories. Beta-diversity of the data set was calculated using the weighted UniFrac metric (Lozupone et al., 2006), which utilizes phylogenetic information, using a re-sampling size of 8500. Principal coordinate plots of the UniFrac distance matrices were then generated to investigate the relationships between microbial communities in each of the samples. Statistical testing for the logarithm of *Campylobacter* count data by farm was carried out using one-way ANOVA. Testing for the difference in microbial community composition between farms was carried out using the DirtyGenes test (Shaw et al., unpublished). A test for the difference in the percentage of Proteobacteria isolated from farms was conducted using non-parametric ANOVA (Kruskal–Wallis test) as the data were not normally distributed (D’Agostino & Pearson normality test). Pair-wise, between-farm comparisons were conducted using Dunn’s multiple comparisons test (GraphPad Prism version 7.03 for Windows, GraphPad Software, La Jolla, CA, United States^4^). *p*-Value of 0.05 was used as cut off for statistical significance.

### Data Availability

The sequencing reads (fastq files) have been submitted to the European Nucleotide Archive, with project number PRJEB23657. The individual reads of samples 1–100 (Supplementary Table 1) are available under accession numbers ERR2206851–ERR2206950, respectively.

## Results

After filtering the data for base-calling quality and amplicon length, 4.86 million sequences were retained for further analysis with a mean of 40,475 (*SD*: 12,478; minimum: 4007; maximum: 77,165) sequences per sample. After clustering with a similarity cut off of 97% these sequences were assigned to 96,553 OTUs. Alpha and beta-diversity was assessed using an evenness of 8,500 sequence reads per sample (maximizing the sample size whilst retaining the maximum number of samples in the analysis), which excluded a single sample from further analysis. Thus, the microbial diversity in the caeca of 99 birds was compared.

### Community Composition of the Caecal Microbiomes and Significant Inter-Farm Variability

**FIGURE 1:**
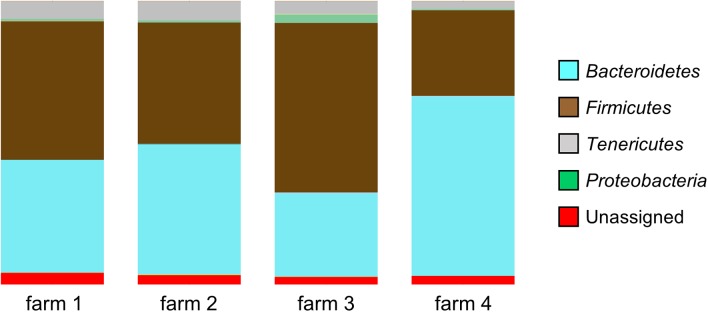
Microbial diversity at phyla level of the samples coming from four different farms showing significant differences in community composition (*p* < 0.001, DirtyGenes test). The percentage of Proteobacteria from Farm 4 was significantly lower than from the other farms (*p* < 0.0001, Kruskal–Wallis test).

According to the sequence data, the caecal microbiome consists mainly of three phyla of bacteria: Bacteroidetes, Firmicutes, and Tenericutes. The proportions of these phyla in the samples varied according to the farm of origin: Bacteroidetes (29.7–63.5%, *SD*: 14.2, and mean: 44.8), Firmicutes (30.2–59.8%, *SD*: 12.3, and mean: 45.4) and Tenericutes (2.8–6.8%, *SD*: 1.3, and mean: 5.1). The inter-farm variability is confirmed by there being a significant difference in the microbial community composition between the four farms (*p* < 0.001), as determined by the DirtyGenes test (Shaw et al., unpublished). In the samples coming from one of the farms (Farm 4), a significantly lower mean percentage (0.3%, range: 0 to 6.2%) of Proteobacteria was observed (**Figure 1**), compared with the mean percentage of this phylum in the other farms (0.8–3.0%, range: 0 to 25.4%), *p*-value < 0.0001.

### High Variability of *Campylobacter* Counts Both Within and Between Farms

**FIGURE 2:**
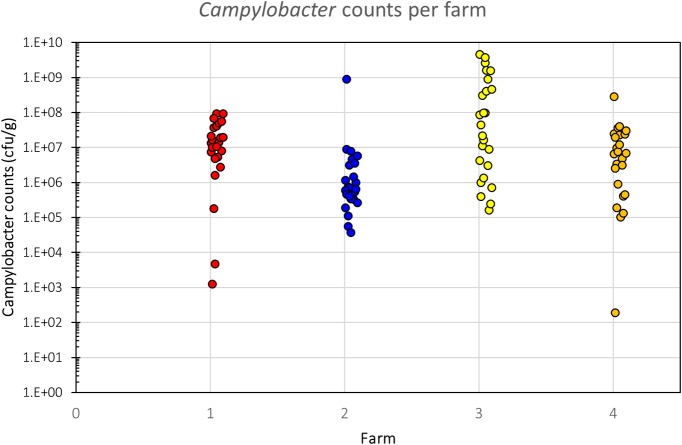
*Campylobacter* counts (CFU g^-1^) of individual birds per farm. There is a significant difference in *Campylobacter* counts between the farms (*p* = 0.00016, one-way ANOVA), despite considerable intra-farm variability.

All samples analyzed were culture-positive for *Campylobacter* spp. (**Figure 2**). There was considerable intra-farm variability between individual birds (2 to 9 log_10_ CFU g^-1^), and the inter-farm variability was also significant (*p* = 0.00016; one-way ANOVA). The samples coming from Farm 3 (samples 26–50) had the highest load of *Campylobacter* with five samples having more than 9 log_10_ CFU g^-1^ and further six having more than 8 log_10_ CFU g^-1^, when all other samples had lower loads and only two samples from the other farms had more than 8 log_10_ CFU g^-1^. Farm 3 also had the highest average bird weight, comparing all chickens that were slaughtered at roughly the same age (data not available for the chickens of Farm 4). The age of the chickens at the time of slaughter and their mean weights are shown in **Table 1**.

### High *Campylobacter* Counts Are Associated With the Presence of Proteobacteria

**FIGURE 3:**
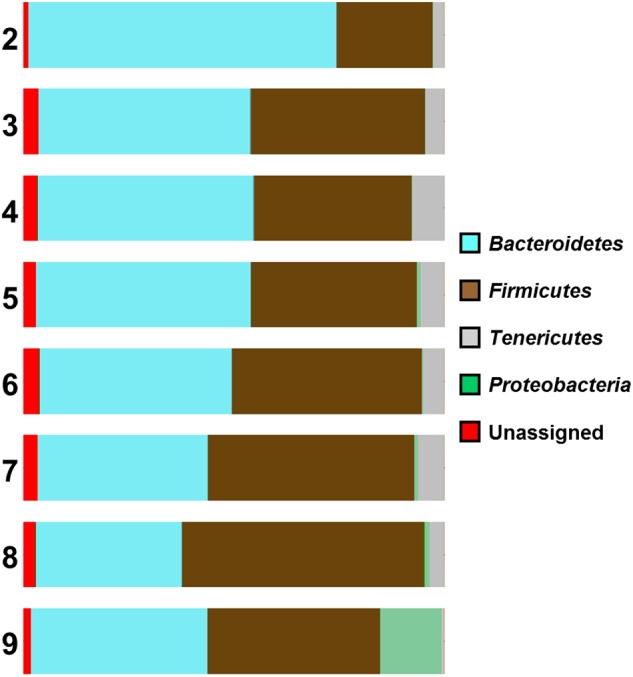
Microbial diversity at phyla level of the samples according to their *Campylobacter* (log) counts (2 log CFU g^-1^ to 9 log CFU g^-1^).

An association between *Campylobacter* counts and the composition of the caecal microbiome was also observed in our study. Analysis of the distribution graphs (**Figure 3**) indicates an increasing proportion of Firmicutes and Proteobacteria, and a decreasing proportion of Bacteroidetes with increasing levels of *Campylobacter*. There is no clear trend for the proportion of Tenericutes, although samples with the highest *Campylobacter* counts (>9 log_10_ CFU g^-1^) had the lowest percentage of Tenericutes (0.62%), whereas the percentage of Proteobacteria is increased (14.56%) (**Figure 3**). The vast majority of these Proteobacteria (91%) belong to the Enterobacteriaceae family, which suggests that *Campylobacter* spp. colonization of the caeca could be associated with a change of their microbiota and that high counts of *Campylobacter* spp. could be linked with a high proportion of Enterobacteriaceae. Furthermore, the number of reads for Epsilonproteobacteria were also higher for chickens highly colonized by *Campylobacter* (>9 log_10_ CFU g^-1^).

**FIGURE 4:**
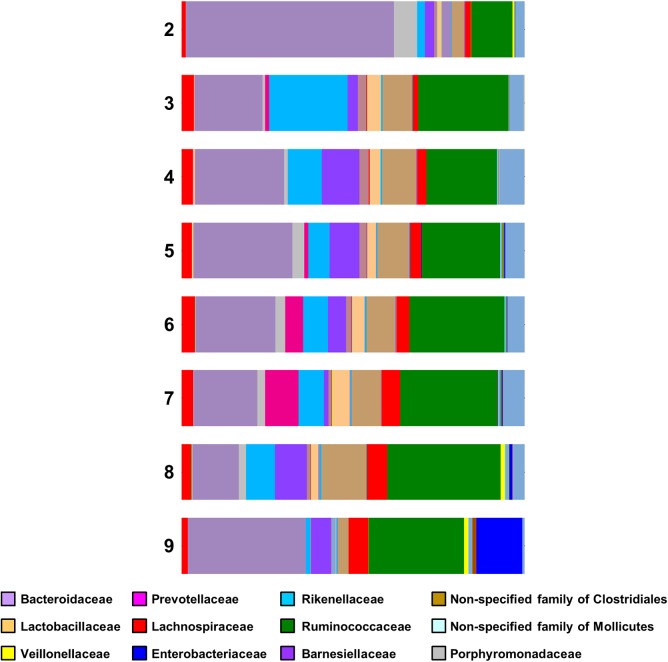
Family bar plot of the microbiome of chicken caeca samples divided according to their *Campylobacter* load (2 log CFU g^-1^ to 9 log CFU g^-1^).

For Lactobacillaceae the percentages are almost constant (2.46–4.89%) for the samples having *Campylobacter* counts from 3 to 7 log CFU g^-1^. There was a small decrease (2.07%) in samples having 8 log CFU g^-1^ and a considerable decrease (0.22%) for the samples having 9 log CFU g^-1^ (**Figure 4**). A statistical test on the differences in microbial communities between birds with the highest *Campylobacter* loads and the other birds would not give reliable results because the comparison is *post hoc*. However, the results do suggest that very high *Campylobacter* loads could be associated with low Lactobacillaceae loads, a hypothesis that could be tested in future studies.

### Inter-Farm Variation Is Greater Than Intra-Farm Variation

**FIGURE 5:**
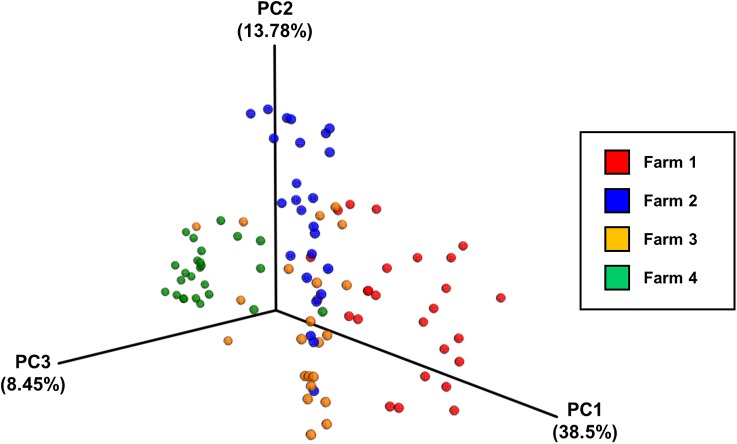
The principal coordinates analysis (PCoA) plot of all samples divided by farm, based on pairwise weighted uniFrac distances between samples.

**FIGURE 6:**
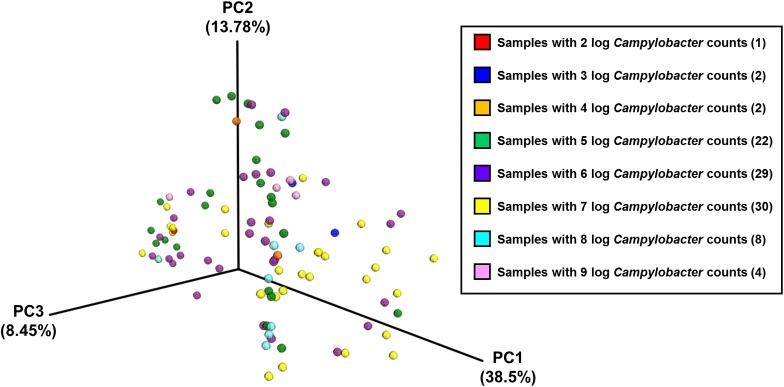
The PCoA plot of all samples divided by *Campylobacter* counts (CFU g^-1^), based on pairwise weighted uniFrac distances between samples.

Analysis of community composition of the samples indicated that inter-farm variation was greater than intra-farm variation (**Figure 5**). Intra-farm variation was more obvious in Farm 3, which was the farm with the highest average weight of the birds of roughly the same age (the birds from Farm 4 were 5 days older at slaughter and their weights not recorded) and with the highest load of *Campylobacter*. The samples from Farm 4 were found to be quite closely related and their diversity was considerably lower than the other farms. In contrast, the samples from Farm 3 were found to have the highest diversity in their microbiota which potentially could be associated with their high load of *Campylobacter* and increased weight. **Figure 6** depicts the principal coordinates analysis (PCoA) of all samples labeled with *Campylobacter* counts (CFU g^-1^). Three out of four samples with the highest *Campylobacter* loads (9 log CFU g^-1^) cluster tightly together except for one sample that differs slightly. The pattern is similar for the other samples having high *Campylobacter* counts (8 log CFU g^-1^), while the samples with lower counts (less than 7 log CFU g^-1^) tend to be more diverse.

## Discussion

The microbiome of the ceacal samples consisted mainly of three phyla of bacteria: Bacteroidetes, Firmicutes, and Tenericutes and the proportions of these phyla varied substantially according to the farm of origin. In two recent studies, the microbial compositions of chicken caeca were reported as consisting of Firmicutes (22–81%), Proteobacteria (5–64%), and Bacteroidetes (1–30%) (Sofka et al., 2015) and Firmicutes (58.9%), Bacteroidetes (25.7%), and Proteobacteria (10.7%) (Mancabelli et al., 2016), respectively. The differences observed in these studies and ours may be due to a number of differing factors including environmental (location and diet), host (host genetics and age), and possibly levels of *Campylobacter*, all of which may influence the microbiota profiles of the birds.

The presence of *Campylobacter* in all samples in our study precluded a simple comparison between the caecal microbiomes of *Campylobacter* positive and negative birds. In a recent study (Sofka et al., 2015), it was found that the proportion of these phyla (Firmicutes, Proteobacteria, and Bacteroidetes) in different samples varied substantially according to the presence of *Campylobacter*, though without statistical significance. A tendency was seen for *Campylobacter* negative samples to have higher amounts of Firmicutes, whereas a higher proportion of Proteobacteria and Bacteroidetes was found in *Campylobacter* positive samples. In our study, the birds from the farm with the highest *Campylobacter* counts had the highest percentage of *Firmicutes* and the lowest percentage of Bacteroidetes, which is the opposite of the findings from the previous study. However, the percentages of Proteobacteria were increased in both studies in birds with the highest counts of *Campylobacter*. Plots of the proportions of Firmicutes, Bacteroidetes, Tenericutes, and Gammaproteobacteria with the *Campylobacter* cfu counts or the proportion of Epsilonprotebacterial reads showed no correlation with the first three categories, but a clear correlation with the last category (Sofka et al., 2015). Such links for the Enterobacteriaceae were previously reported (Bereswill et al., 2011; Dicksved et al., 2014; Sofka et al., 2015), but it is not clear whether higher levels of *Campylobacter* create conditions beneficial for Enterobacteriaceae, or vice versa.

A study by Singh et al. (2013) suggested that Firmicutes to Bacteroidetes ratio based on 16S rRNA sequencing could be correlated with the weight of the birds and antibiotic treatment. A further study also suggested that antibiotic treatment altered gut and fecal bacterial species composition in chickens toward an increased abundance of *Lactobacillus* spp., Clostridiales and Enterobacteriaceae (Gong et al., 2008). These findings were partially confirmed by our study. Farm 3 was the farm with the highest ratio of Firmicutes to Bacteroidetes and the chickens taken from that farm were the ones with the highest weight. Unfortunately, no information was provided for the antibiotic use in this farm. However, the percentages of Clostridiales and Enterobacteriaceae gradually increased as the *Campylobacter* counts of the samples increased: Enterobacteriaceae were highest (13.3%) when the *Campylobacter* counts reached 9 log_10_ CFU g^-1^. For Clostridiales, their peak (53.99%) was in samples having 8 log_10_ CFU g^-1^ of *Campylobacter* counts and they presented a small decrease (38.33%) for the samples having 9 log_10_ CFU g^-1^ (data not shown).

For Lactobacillaceae, the percentages were highly consistent for all samples apart from a noteworthy decrease observed in samples having greater than 9 log_10_ CFU g^-1^. This is in line with previous studies. For example, a study in mice demonstrated that a diet-induced alteration of the intestinal microbiota, comprising an increase in the abundance of *E. coli* and a decrease in *Lactobacillus* was associated with a greater susceptibility to *C. jejuni* infection (Bereswill et al., 2011). Furthermore, another study reported an association between higher *C. jejuni* counts and lower abundance of *Lactobacillus* (Kaakoush et al., 2014). The same research group showed that not only *Lactobacillus* spp. were lower in abundance but *Escherichia* was a major contributor in chickens colonized with *C. jejuni*, since an increase in the abundance of *E. coli* and a decrease of *Lactobacillus* spp. were associated with a greater susceptibility to *C. jejuni* infection.

Recent studies (Hilbert et al., 2010; Haag et al., 2012; Dicksved et al., 2014; Kaakoush et al., 2014) have shown that *Campylobacter* closely interacts with other microorganisms, the intestinal microbiota is important for *Campylobacter* colonization in chickens (Kaakoush et al., 2014) and *Campylobacter* colonization leads to a change in the intestinal microbiota (Haag et al., 2012). According to another recent study (Sofka et al., 2015), the caecal microbiota in *Campylobacter*-free and *Campylobacter* colonized chickens differed considerably. The cultural colony counts of lactic acid bacteria, Enterobacteriaceae, *E. coli* and total aerobic counts were found to be significantly higher in *Campylobacter* negative samples. The absence of *Campylobacter*-free samples didn’t allow our research to corroborate this. However, as mentioned previously, by taking into account the samples with the high and low *Campylobacter* loads, we can partially corroborate the finding since Lactobacillaceae were lower in abundance when *Campylobacter* load was more than 8 log_10_ CFU g^-1^ and Enterobacteriaceae reached its highest percentage for the samples having more than 8 log_10_ CFU g^-1^ *Campylobacter* load. Nevertheless, as the high-yield *Campylobacter* samples (more than 9 log CFU g^-1^) were all from the same farm, it is possible that there are confounding factors.

The PCoA of the data indicated that there was more between-farm variation in the complete microbial community composition than within-farm variation. The use of weighted uniFrac metrics to determine the distances between samples reveals differences that are due to changes in relative taxon abundance. Given the close proximity of the birds on a farm, the convergence of their caecal microbiomes is not surprising. Differences between farms could be linked to environment, diet or even the age of the birds.

The outcomes of the present study suggest that there is strong evidence that high counts of *Campylobacter* in broiler caeca are interrelated with the microbial community structure and particularly associated with an increase in the presence of Enterobacteriaceae. However, further studies need be done to establish causality and to investigate whether the presence of *Campylobacter* favors the growth of Enterobacteriaceae or vice versa. It is possible that a general dysbacteriosis leads to colonization by both *Campylobacter* and Enterobacteriaceae. Furthermore, it was found that a decrease in *Lactobacillus* abundance in chicken caeca could be linked to high *Campylobacter* loads. Therefore, it can be concluded that *Campylobacter* colonization might be associated with changes in the intestinal microbiota and the strategies to reduce colonization of *Campylobacter* in chickens could include shaping the chicken microbiota. The study of the chicken microbiota is considered necessary to provide new and effective approaches to fight *Campylobacter* colonization and improve public health.

## Author Contributions

IS was the PI and wrote the manuscript. RE did the sequencing analysis and wrote parts of the manuscript. SC did the microbiological analysis and wrote parts of the manuscript. AV helped with the analysis of the data and wrote parts of the manuscript. DS did the statistical analysis of the data. JP helped with the design of the study and reviewed the manuscript. MC helped with the design of the study, wrote parts of the manuscript, and reviewed it. RLR helped with the design of the study and reviewed the manuscript. AC had the initial idea of this study, helped with the design of the study, and reviewed the manuscript.

## Conflict of Interest Statement

The authors declare that the research was conducted in the absence of any commercial or financial relationships that could be construed as a potential conflict of interest.

## Footnotes

1 https://github.com/mlangill/microbiome_helper/

2 http://sco.h-its.org/exelixis/web/software/pear/

3 http://greengenes.secondgenome.com/

4 www.graphpad.com
