# Supplementary material for: Investigating the Association Between the Caecal Microbiomes of Broilers and Campylobacter Burden
Source: Front Microbiol. 2018 May 22;9:927. doi: 10.3389/fmicb.2018.00927 (PMC5972209; doi:10.3389/fmicb.2018.00927)
Supplement: Supplementary file 1 [file Table_1.DOCX]

**Supplementary material**

Table 1. *Campylobacter* loads (cfu/g) in individual birds.

| Bird ID | *Campylobacter* level (cfu/g) | Bird ID | *Campylobacter* level (cfu/g) | Bird ID | *Campylobacter* level (cfu/g) | Bird ID | *Campylobacter* level (cfu/g) |
| --- | --- | --- | --- | --- | --- | --- | --- |
| 1 | 2.60E+07 | 26 | 8.90E+07 | 51 | 1.22E+06 | 76 | 1.41E+07 |
| 2 | 2.00E+02 | 27 | 1.05E+06 | 52 | 9.30E+06 | 77 | 1.72E+07 |
| 3 | 2.01E+05 | 28 | 1.18E+07 | 53 | 7.80E+05 | 78 | 3.90E+07 |
| 4 | 3.80E+07 | 29 | 1.40E+06 | 54 | 7.50E+05 | 79 | 1.70E+06 |
| 5 | 2.40E+07 | 30 | 2.71E+09 | 55 | 8.20E+06 | 80 | 9.80E+07 |
| 6 | 3.60E+06 | 31 | 1.68E+09 | 56 | 3.80E+06 | 81 | 1.33E+07 |
| 7 | 5.00E+06 | 32 | 9.30E+08 | 57 | 3.50E+05 | 82 | 1.46E+07 |
| 8 | 4.30E+05 | 33 | 1.70E+05 | 58 | 5.70E+05 | 83 | 2.90E+06 |
| 9 | 2.50E+07 | 34 | 1.66E+09 | 59 | 1.04E+06 | 84 | 8.40E+06 |
| 10 | 7.20E+06 | 35 | 7.50E+05 | 60 | 2.80E+05 | 85 | 9.80E+07 |
| 11 | 6.80E+06 | 36 | 4.80E+09 | 61 | 6.30E+05 | 86 | 2.20E+07 |
| 12 | 2.04E+07 | 37 | 4.20E+05 | 62 | 9.30E+08 | 87 | 1.30E+03 |
| 13 | 3.50E+06 | 38 | 3.20E+08 | 63 | 5.80E+04 | 88 | 1.91E+05 |
| 14 | 9.40E+05 | 39 | 1.71E+07 | 64 | 3.30E+06 | 89 | 4.90E+03 |
| 15 | 4.20E+07 | 40 | 1.01E+08 | 65 | 3.60E+05 | 90 | 4.30E+07 |
| 16 | 1.09E+05 | 41 | 4.30E+08 | 66 | 4.80E+06 | 91 | 5.60E+06 |
| 17 | 3.30E+06 | 42 | 3.20E+06 | 67 | 1.51E+06 | 92 | 5.30E+07 |
| 18 | 1.39E+05 | 43 | 9.40E+06 | 68 | 3.70E+06 | 93 | 2.00E+07 |
| 19 | 4.70E+05 | 44 | 2.60E+05 | 69 | 6.80E+05 | 94 | 5.80E+07 |
| 20 | 3.20E+07 | 45 | 4.80E+08 | 70 | 6.10E+06 | 95 | 2.06E+07 |
| 21 | 3.00E+08 | 46 | 4.50E+06 | 71 | 2.00E+05 | 96 | 7.90E+06 |
| 22 | 2.70E+06 | 47 | 4.70E+07 | 72 | 4.90E+05 | 97 | 1.07E+07 |
| 23 | 1.01E+07 | 48 | 2.30E+07 | 73 | 1.18E+05 | 98 | 7.20E+07 |
| 24 | 8.00E+06 | 49 | 1.03E+08 | 74 | 4.30E+05 | 99 | 5.10E+06 |
| 25 | 1.26E+07 | 50 | 3.90E+09 | 75 | 3.90E+04 | 100 | 1.11E+07 |

Farms 1, 2, 3 and 4: Samples 76-100, 51-75, 26-50, and 1-25, respectively.
